# The relationship between maternal employment and stunting among 6–59 months old children in Gurage Zone Southern Nation Nationality People’s region, Ethiopia: A comparative cross-sectional study

**DOI:** 10.3389/fnut.2022.964124

**Published:** 2022-10-06

**Authors:** Mekiya Ahmed, Kebebush Zepre, Kifle Lentero, Tigist Gebremariam, Zeyneba Jemal, Asegedech Wondimu, Jemal Bedewi, Tamirat Melis, Alazar Gebremeskel

**Affiliations:** ^1^Maternal, Neonatal and Child Health and Nutrition Core Process, Gurage Zone Health Department, Wolkite, Ethiopia; ^2^Department of Public Health, College of Medicine and Health Science, Wolkite University, Wolkite, Ethiopia; ^3^College of Medicine and Health Science, Hawassa University, Hawassa, Ethiopia

**Keywords:** stunting, employment, associated factor, children, Gurage Zone, Ethiopia

## Abstract

**Background:**

Motivating proper nutrition during childhood is the basis for optimal health, learning, productivity, and social wellbeing throughout life. Stunting is among the major public health problems. According to the Ethiopian mini demographic and health survey, the prevalence of stunting among under five children was 37%. In addition, stunting has a trans-generational effect on a mother’s nutritional status. However, evidence on the causal contribution of maternal employment to stunting among under five children is not well understood in Ethiopia. This study aimed to compare the stunting status and associated factors among under five children of employed and unemployed mothers in the Gurage Zone, Southern Ethiopia, in 2021. A community-based comparative cross-sectional study was conducted among 671 (330 employed and 341 unemployed) randomly selected mother–child pairs in the Gurage Zone, Southern Ethiopia. A pretested semi-structured tool and validated anthropometric measurements were used to collect the data. The data were entered into Epi Data version 3.1 and exported to Statistical Package for Social Science (SPSS) version 23.0 for analysis. Frequency, percent, mean, median, and *SD* were computed and presented by using tables and figures. A bivariable and multivariable binary logistic regression analysis was conducted to assess the association between factors and outcome variables.

**Results:**

In this study, a total of 671 mother–child pairs (330 (94.60%) employed and 341 (97.70%) unemployed) participated, with a total response rate of 96%. Among the total participants, about 70 (21.2%) [95% CI: (17.0, 25.5)] and 98 (28.8%) [95% CI: (23.0, 33.4)] of children of employed and unemployed mothers, respectively, were stunted. Mothers’ level of education, primary and secondary [AOR = 1.79, 95% CI: (0.8, 3.7), age between 25 and 29 years [AOR = 0.08, 95% CI: (0.006, 0.904)], monthly family income > 5,000 birr [AOR = 0.42, 95% CI: (0.00, 0.64)], and children aged between 6 and 23 months [AOR = 2.9; 95% CI: (1.48, 5.80)] were predictors of stunting among the children of employed mothers. Compared to the mothers who did not receive nutritional education [AOR = 2.5; 95% CI: (1.10, 5.60)], monthly family income of 2,000 ETB [AOR = 2.64; 95% CI: (1.34, 5.19)], sex of child (girl) [AOR = 2.3; 95% CI: (1.30, 3.80), and mothers educational status of read-and-write only [AOR = 2.9, 95% CI: (1.40, 5.80)] were predictors of stunting among the children of unemployed mothers. The nutrition intervention should focus on encouraging women’s education as it increases the probability of being employed, improving the income of families by using different income-generating strategies, and strengthening the existing essential nutrition counseling strategy. Likewise, further research work on the difference between employed and unemployed mothers on stunting status is also recommended to researchers.

## Introduction

Stunting is defined as a long-term lack of adequate nutrition in children, which limits linear growth and leads to collective growth deficits (1–3). It is the most common type of undernutrition and is regarded as a major public health issue in the country (4, 5). Stunting is an anthropometric gauge regularly used to compute a child’s long-term nutritional condition. It would be defined as *Z*-scores of less than 2 SDs of height for age (6–9). Proper nutrition is crucial for healthy growth and development. It ensures proper organ formation and function, a strong immune system, and neurological and cognitive development. It is also the foundation of survival. The effect of proper nutrition during childhood is trans-generational (not only in early life but also across adulthood) (7).

Food insecurity, inadequate maternal and child care, poor health services, and living circumstances, or improper feeding practice cause measurable unfavorable effects on body function and clinical outcomes (10–12). There are various causes of stunting; these causes are versatile and tangled with each other, hierarchically related and intergenerational. Stunting among children depends on the multifaceted interactions of various factors such as socio-demographic, environmental, reproductive, institutional, cultural, political, and regional (13, 14).

Because of the intergenerational crash of chronic malnutrition, childhood stunting is connected with the poor-nutritional status of future mothers and an advanced risk of death (15). In low-income settings, micronutrient consumption of women is insufficient due to the limitation of resources, and it increases the risk of child mortality (16). As a result, women’s employment has a positive effect because it increases household income, which improves household nutrition in general and the nutritional status of women in particular (17). In addition to the multiple roles of women in the health and welfare of all family members, women’s employment has a direct effect on child care, the nutritional status of children, and the mother herself (10, 18, 19).

In 2018, 22% of under five children were stunted globally (20). In developing countries, one out of four children are stunted (3, 18). Ethiopia has the second highest rate of stunting in the Sub-Saharan Africa (19). Worldwide, 8–11 million under five children die each year (8, 20–22). A total of 45% of these deaths are attributed to stunting, which is mostly avoidable through economic development and public health measures (3, 12, 14, 19, 23, 24).

According to the 2019 Ethiopia, mini demographic and health survey (EMDHS), 37% of under-five children are short for their age or stunted and 12% are severely stunted. The prevalence of stunting generally increases gradually with age, from 22% among children aged 6–8 months old up to 44% of children aged 48–59 months, and it is also slightly higher among boys than in girls (40% vs. 33%) (4).

According to the World Bank estimate, in Ethiopia, 16% of all repetitions in primary school are connected to stunting. Stunted children are delayed by 1.1 y in school education (25, 26). A study from Ethiopia provides evidence that due to children undernutrition, Ethiopia loses an estimated 55.5 billion Ethiopian birr each year. This is equivalent to 16.5% of the GDP. There were an estimated 4.4 million additional clinical episodes associated with undernutrition in children under the age of five at a cost of ETB 1.8 billion (27).

Stunting in children is declining with much effort made by various stakeholders. However, Ethiopia is not on track to realize the national nutrition program (NNP) II target and the Sequota declaration to reduce stunting at the rate of 26% by 2020. Besides, there is a great disparity in stunting rates across regions, residences, and economic status. Hence, stunting remains a public health concern in Ethiopia and more intervention is considered necessary to accelerate learning (28). Involving women in income-generating activities such as employment increases household income, with the resultant advantage of increasing women’s status and power of decision-making (14).

In addition, there is an agreement that income earned by a mother or maternal employment has a direct effect on childcare, the nutritional status of children, and the mothers themselves (10). Furthermore, maternal employment empowers women economically and socially, and is in line with sustainable development goal 8, which aims at promoting economic growth and productive employment for all. In addition, sustainable development goal 2 aims at ending hunger, achieving food security, and improving nutrition (20).

Although previous literature shows that a high risk of stunting is associated with socioeconomic classes (14), there is a dearth of literature connecting maternal employment with childhood stunting in Ethiopia, which needs to be supported by solid evidence. Thus, this study is aimed at understanding the relative contributions of maternal employment to childhood stunting.

## Materials and methods

### Study setting and design

This community-based comparative cross-sectional study was conducted in the Gurage Zone, 158 km away from Addis Ababa, the capital city of Ethiopia, and 345 km from Hawassa, the capital city of the Southern Nation Nationality People’s region (SNNPR). In the district, there are three sub-cities and seven kebeles (which refer to the lower-level administrative divisions) under these sub-cities. It has a total population of 72,929 living within 14,585 households. A total of 11,377 of them were estimated to be under five children, and 9,772 were aged between 6 and 59 months. The study was conducted in May 2021, in the Gregorian calendar.

### Population and eligibility criteria

The findings of this survey are projected for all mother—child pair aged 6–59 months in the Gurage Zone, while those children aged 6–59 months from a randomly selected kebele were the study population. Those children aged 6–59 months old who have resided in the study area for at least 6 months and above and who have volunteered to participate in the study were considered as study participants, while those children whose mothers were unable to respond to the interview due to severe illness were excluded. Sample size determination.

The sample size was determined by considering both objectives; for the first objective, we assumed the prevalence of stunting (P) among children of unemployed and employed mothers was 26.5 and 18.5%, respectively, from previous studies (18), *f* () = 7.84, when the power = 80%, and = 5% considering a 10% non-respondent rate and a 1.5 design effect, using a statistical formula.

$$\eta =\left(p1q1+p2q2\right)f\left(\left(\alpha ,\beta \right)\right)/({\left(p1-p2\right)}^{2}$$

yield⁢sample⁢size⁢of⁢698.

While the sample size for the second objective was estimated using the sample size for a cross-sectional study to compare the factor variable with stunting under Epi-info version 7 software. Assumptions were taken into consideration; mothers’ educational status (14), mothers’ employment status (18), and sex of children (29) as factors; confidence level = 95%, power (1-): 80%, ratio = 1:1 (employed and unemployed), and a design effect of 1.5, giving up the largest sample size of 647 for unemployed mothers at an adjusted odds ratio (AOR) of 2, which is less than that calculated by the double population proportion formula. Therefore, the final sample size was 698 (349 employed and 349 unemployed).

### Sampling procedures

**FIGURE 1:**
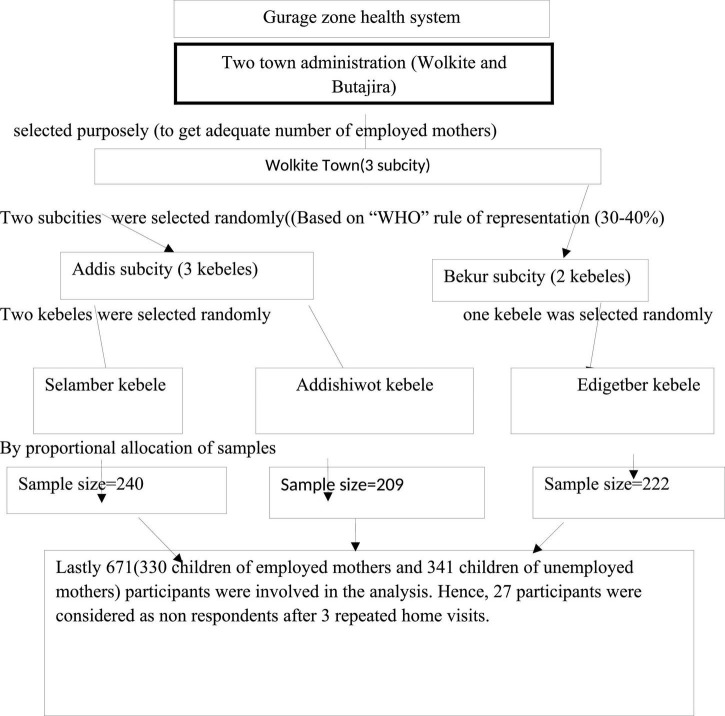
Schematic presentation of the sampling procedure for a study on the relationship between maternal employment and stunting among 6–59-month-old children in Gurage Zone SNNPR, Ethiopia 2021.

A multi-stage sampling technique was used to randomly select 698 child–mother pairs from a randomly selected household. The sample size was proportionally allocated to randomly selected sub-cities. Then, from each sub-city, a pre-specified number of kebeles were selected, and the sample was further allocated proportionally to each randomly selected kebele. A total of 3 kebele were included [Based on the “WHO” rule of representation (30–40%)] (see Figure 1).

After that, to select the allocated sample of participants from each kebele, we applied systematic random sampling. The sample frame containing the full list of households with children aged 6–59 months was obtained from the list (registered 2 weeks prior to the actual data collection process for this purpose). When there were two 6–59-month-old children in a household, one was selected randomly by lottery method. When the respondent was not available at the time, the data collectors made about 3 visits.

### Data collection tools and procedure

A pretested structured interviewer-administered questionnaire containing variables assessing socio-demographic variables, household economic status, maternal health status, and child health-related factors, was used to collect data from the mother or caregiver of the child. After reviewing relevant literature from prior related studies, the questionnaire was prepared in English and translated to the local language (Amharic), and administered in translated form. The questionnaire was pretested a week before the actual survey in an analogous setting in Butajira town on 5% of the calculated sample size and an amendment of the tool was done based on the result from the pretest. After interviewing the mothers of the selected children, the anthropometric measurement for height/length was measured in accordance with WHO guidelines. A portable stand-meter (SECA) was used to measure older children (older than 2 years) while younger children (less than 2 years) were measured using a calibrated length board. The body parts were in touch with the measuring board in proper anatomical position. The child’s body at the occiput, shoulder blades, buttocks, and heels were in touch with the board and recorded to the nearest 0.1.

### Variables of the study

The dependent variable of this study is stunting. Maternal health and health service-related factors such as antenatal care (ANC) follow-up, nutritional information, child health-related factors such as breastfeeding practice, meal frequency, immunization status, and diarrhea exposure.

### Quality control of data

The questionnaire was pretested and administered in the local language. An adequate number of data collectors and supervisors were employed for actual data collection and trained on how they proceeded, and close supervision was undertaken throughout the process. The completeness and consistency of the collected data were checked on a daily basis.

### Operational definitions/measurement/

**Stunted—**Children whose height-for-age *Z*-score is below minus two standard deviations (-2 *SD*) from the median of the WHO standard (16, 20).

### Definition of terms

A caregiver is the most responsible person that provides child care when the mother is out of the home for work. A mother is considered to be an “employed mother” if she reports earning income since the birth of the current eligible child until the data collection period by working either in a government, NGO, public, private sector, or other self-managed income-generating work. A mother is considered “unemployed” if she reports having had no work since the birth of her current eligible child until the data collection period, whether in the government, NGO, public, or private sector, or from other self-managed income-generating work.

### Processing and analysis of data

The data were coded and entered into the statistical software Epi Data version 3.1 before being exported to the Statistical Package for Social Science (SPSS) version 23.0 for analysis. The analysis was done; frequency, percent, mean, median, and standard deviation were computed, and presented by using tables and figures. A bivariable and multivariable binary logistic regression analysis was conducted to assess the association between the factors and outcome variables. The variables were checked for normality, and multicollinearity (using statistically significant correlations and a higher variance inflation factor above 10). Factors with a *p*-value below 0.20 in bivariate were considered for the final logistic regression model to address all possible confounders. Model fitness was tested by using the Hosmer-Lemeshow goodness-of-fit (0.689 and 0.446) for employed and unemployed, respectively, which were good fits for the data). Crude and adjusted odd ratios with 95% confidence intervals were reported. Independent predictors were declared at a *P*-value below 0.05.

### Ethical considerations

Ethical clearance was obtained from the Institutional Health Research Ethical Review Committee at Wolkite University. An official support letter was submitted to those who are concerned. The study, its purpose, procedure, and duration, the rights of the respondents, and possible risks and benefits of the study were clearly explained to each participant using the local language. At last, written informed consent was obtained from participants before they participated in the study. All COVID-19 pandemic prevention standard precautions were followed, including data confidentiality.

## Results

### Socio-demographic and economic characteristics

This study was undertaken among 671 mothers (330 (94.6%) employed and 341 (97.7%) unemployed) who have children aged 6–59 months in southern Ethiopia, with a total response rate of 96%. The respondents’ average age was 32.1 years (+ 5.8), and the age range was 24–45. A total of 301 (91.2%) employed and 326 (95.6%) unemployed were married; 148 (44.8%) employed and 151 (44.3%) unemployed were Muslim; 130 (39.4%) employed and 145 (42.5%) unemployed were Orthodox; 192 (58.2%) employed and 49 (14.4%) unemployed mothers had attended college or higher; and 208 (63%) and 267 (78.3%) employed and unemployed mothers had family sizes of 4–6. More than half (59%) of the employed mothers had a family income of > 5,000 ETB per month, whereas around two-thirds (68.3%) of the unemployed mothers earn between 2,000 and 5,000 ETB (see Table 1).

**TABLE 1:** Socio-demographic characteristics of mothers who have 6–59 months old children, in Gurage Zone SNNPR Ethiopia, 2021 (*n* = 671).

Variables	Employed (*N* = 330)	Unemployed (*N* = 341)	Total (*N* = 671)
	Count (%)	Count (%)	Count (%)
**Maternal age**			
20–24 years	9 (2.73%)	5 (1.46%)	14 (2.08%)
25–29 years	127 (38.50%)	122 (35.78)	249 (37.11%)
30–34 years	81 (24.53%)	80 (23.6%)	161 (24%)
≥ 35	113 (34.24)	134 (39.3)	247 (36.81)
**No of family**			
1–3	14 (4.2)	5 (1.5%)	19 (2.8%)
4–6	208 (63%)	267 (78.3%)	475 (70.8%)
>6	108 (32.7%)	69 (20.2%)	177 (26.4%)
**Marital status**			
Marred and live together	301 (91.2%)	326 (95.6%)	627 (93.4%)
Divorced	14 (4.2%)	4 (1.2%)	18 (2.7%)
Widowed	11 (3.3%)	9 (2.6%)	20 (2.98%)
Never marred	4 (1.2%)	2 (0.6%)	6 (0.9%)
**Religion**			
Orthodox	130 (39.4%)	145 (42.5%)	275 (41%)
Muslim	148 (44.8%)	151 (44.3%)	299 (44.2%)
Catholic	14 (4.2%)	19 (5.6%)	33 (4.9%)
Protestant	38 (11.5%)	26 (7.6%)	64 (9.5%)
**Family income**			
<2,000 ET birr	60 (18.2%)	108 (31.7%)	166 (24.7%)
2,000–5,000 ET birr	74 (22.4%)	233 (68.3%)	313 (46%)
>5,000 ET birr	196 (59.4%)	0 (0%)	192 (28.6)
**Educational status**			
Read and write	46 (13.9%)	90 (26.4%)	136 (20.27%)
Primary to secondary	100 (30.3%)	202 (59.2%)	302 (45.0%)
College and above	184 (55.8%)	49 (14.4%)	233 (34.73%)
**Educational status of husband’s**			
Read and write	10 (3.0%)	2 (0.6%)	12 (1.8%)
primary to secondary	93 (28.2%)	210 (61.6%)	303 (45.2%)
College and above	227 (68.8%)	129 (37.8%)	356 (53.1%)

### Characteristics of 6–59-month-old children of employed and unemployed mothers

Among the total 154 (46.7%) children of employed and 160 (46.9%) children of unemployed mothers, 105 (31.8%) children of employed and 97 (28.4%) children of unemployed mothers were aged 6–23 months, whereas 255 (68.2%) children of employed and 244 (71.6%) children of unemployed mothers were aged 24–59 months. In total, 326 (98.8%) children of employed and 332 (93%) children of unemployed mothers were vaccinated for age. Regarding the feeding patterns of half of the children, 167 (50.6%) and 173 (50.7%) of children of employed and unemployed mothers, respectively, were breastfed eight times per day. In total, 228 (69.1%) and 255 (74.8%) of children of employed and unemployed mothers, respectively, had taken complementary diets three to four times per day.

### Maternal characteristics

Regarding service utilization during pregnancy, all mothers in both groups have reported that they have gotten ANC services at health facilities at least once. A total of 179 (54.2%) employed and 253 (74.2%) unemployed mothers reported visiting a health facility four or more times during their most recent pregnancy. Concerning nutrition information received during any service delivery time, 246 (74.5%) employed mothers and 284 (83.3%) unemployed mothers reported receiving nutrition information. Regarding delivery service, 322 (97.6%) and 324 (95%) of employed and unemployed mothers, respectively, gave birth at the health facility.

### Prevalence of stunting among 6–59-months-old children of employed and unemployed mothers

Among the total participants, about 70 (21.2%) [95% CI: (17, 25.5)] and 98 (28.8%) [95% CI: (23, 33.4)] children of employed and unemployed mothers, respectively, were stunted.

### Factors associated with stunting among children of employed mothers

The association between stunting and factor variables were assessed using a step-wise backward binary logistic regression model. Variables with a *p*-value below 0.2 in the bivariable logistic regression analysis were considered for the multivariable logistic regression model.

In bivariate analysis, the age of mothers, maternal education, husband education, family income, age of the child, frequency of breastfeeding, diarrhea, frequency of ANC visit, and nutrition information were associated with stunting at *p*-value ≤ 0.2 and have been a candidate for multivariate logistic regression analysis. In the multivariate analysis, maternal education, maternal age, child age, family income, and frequency of breastfeeding showed a statistically independent significant association with stunting at a *p*-value < 0.05.

Children whose mothers attended primary and secondary level education [AOR = 1.79, 95% CI: (0.8, 3.7)] were 1.8 times more likely to be stunted compared to children whose mothers attend college and above. Children whose mothers’ age ranged between 25 and 29 [AOR = 0.08, 95% CI: (0.009, 0.648)] were 92% protected from stunting compared to children whose mothers’ age ranged between 20 and 24 (see Table 2). Children from families with a monthly income of 2,000–5,000 ETB [AOR = 0.045, 95% CI: (0.013, 0.155)] and > 5,000 ETB [AOR = 0.042, 95% CI: (0.014, 0.129)] were 95.5 and 95.8%, respectively, less likely to be stunted as compared to children from families with a monthly income of < 2,000 ETB (see Table 2). Children aged 24–59 months [AOR = 0.37, 95% CI: (0.11, 0.78)] were 63% less likely to be stunted compared with children aged between 6 and 23 months. Likewise, children who breastfeed ≥ 8 times per day [AOR = 0.081, 95% CI: (0.027, 0.247)] were 91.9% less likely to be stunted as compared to the children who breastfeed < 8 times per day (see Table 2).

**TABLE 2:** Bivariate and multivariate analysis for stunting among employed mothers in Gurage Zone, SNNPR, Ethiopia 2021 (*n* = 671).

Variables	Category	Stunted		COR [95% CI]	AOR [95% CI]	*P*-value
		Yes	Not			
Maternal education	Read and write	14	32	0.69 (0.34,1.42)	0.14 (0.04,0.55)	≤0.26
	Primary to secondary	13	87	2.04 (1.03,4.01)	1.79 (1.20,3.70)	≤0.03*
	College and above	43	141	1	1	
Monthly family income in ETB	<2,000	8	52	1	1	
	2,000–5,000	12	62	0.79 (0.3,2.0)	0.04 (0.01,0.15)	≤0.00**
	>5,000	50	146	0.45 (0.2,1.0)	0.04 (0.01,0.13)	≤0.00**
Age of child	6–24 months	13	92	1	1	
	24–59 months	57	168	0.42 (0.21,0.80)	0.37 (0.11,0.78)	≤0.01**
Maternal age	20–24 years	6	2	0.74 (0.36,1.48)	0.49 (0.17,1.43)	≤0.19
	25–29 years	24	103	0.75 (0.01,0.39)	0.08 (0.01,0.64)	≤0.02*
	30–34 years	19	62	0.74 (0.51,1.85)	0.65 (0.23,1.85)	≤0.42
	<=35 years	21	93	1	1	
Frequency breast feeding	<8 times per/day	63	104	1	1	
	≥8 times per day	7	156	0.07 (0.03,0.16)	0.08 (0.03,0.25)	≤0.00**
Diarrhea in the last 2 weeks	Yes	5	45	1	1	
	No	65	215	0.37 (0.14,0.96)	0.35 (0.10,1.25)	≤0.11
ANC visit	<4 times	23	128	1	1	
	≥4 times	47	132	0.50 (0.29, 0.88)	0.74 (0.30,1.87)	≤0.54
Nutrition information	Yes	61	185	1	1	
	No	9	75	2.74 (1.29, 5.80)	1.81 (1.90, 5.20)	≤0.46

COR, Crude odds ratio; AOR, Adjusted odds ratio. *p < 0.05, **p ≤ 0.01.

### Factors associated with stunting among children of unemployed mothers

In bivariate analysis maternal education, family income, age of the child, sex of the child, and nutrition education were significantly associated with stunting at a *p*-value of ≤ 0.2 and 95% CI. Those variables significantly associated (*p* ≤ 0.2) were considered as a candidate for multivariate analysis to control the potential confounding variable. In the final model, sex of the child, nutrition information, maternal education, and family income had a statistically significant association with stunting at a *p*-value of < 0.05 and 95% CI (see Table 3).

**TABLE 3:** Bivariate and multivariate analysis for stunting among unemployed mothers in Gurage Zone, SNNPR, Ethiopia, 2021 (*n* = 671).

Variables	Stunted		COR with 95% CI	AOR with 95% CI	*P*-value
	Yes	No			
**Sex of child**					
Male	56	95	1	1	
Female	42	148	2.0 (1.2, 3.3)	2.3 (1.3,3.8)	≤0.01*
**Nutrition information**					
Yes	87	197	1	1	
No	11	46	1.8 (1.3, 3.7)	2.5 (1.1, 5.6)	≤0.02*
**Maternal education**					
Read and write	13	77	3.2 (1.7,6.3)	2.9 (1.4, 5.8)	≤0.01**
Primary to secondary	72	130	1	1	
College and above	13	36	1.5 (1.2, 6.31)	1.4 (01.6, 3.2)	≤0.31
**Monthly family income**					
<2,000 ET birr	41	67	1.88 (1.157, 3.08)	2.64 (1.34, 5.19)	≤0.01**
≥2,000 ET birr	57	176	1	1	
**Age of child**					
6–23	31	66	1	1	
24–59	67	177	1.2 (0.7, 2.0)	1.3 (1.3,3.8)	≤0.12

COR, Crude odds ratio; AOR, Adjusted odds ratio. *p < 0.05, **p < 0.01.

The multivariate analysis revealed that the odds of being stunted were 2.5 times higher [AOR = 2.5, 95% CI: (1.1, 5.6)] among children of unemployed mothers who had no exposure to nutrition education compared with those children of unemployed mothers who had received nutrition education. Children of unemployed mothers whose mothers were only able to read and write [AOR = 2.9, 95% CI: (1.4, 5.8)] were 2.9 times more likely to be stunted compared to children of unemployed mothers whose mothers had attended primary and secondary school. Likewise, children of unemployed mothers whose family monthly income < 2,000 ETB [AOR = 2.64, 95% CI: (1.34, 5.19)] were nearly three times more likely to be stunted as compared with the children whose family monthly income was greater or equal to 2,000 ETB. And a girl child [AOR = 2.3, 95% CI: (1.3, 3.8)] is two times more likely to be stunted compared with a boy (see Table 3).

## Discussion

This article quantified and compared the burden of stunting and its predictors among children of employed and unemployed mothers. A considerable proportion of children of employed and unemployed mothers [21.2%, 95% CI: (17, 25.5) and 28.8%, 95% CI:: (23, 33.4)] were stunted, which showed that the prevalence of stunting is slightly higher among children of unemployed mothers. It is consistent with other study findings from Wolayta Sodo, town administration, Southern Ethiopia (18.5 and 26.5%), respectively (14), and Wilayat Zone, Southern Ethiopia, 21.8 and 22.6%, respectively (18). This might be due to socio-demographic/economic/and cultural similarities between these study areas. However, other survey reports from Adama town, central Ethiopia (2017) (28 and 39.5%), respectively (10) show that the possible reason for this difference could be the time gap between the studies, as an intensive intervention in childhood.

In this study, we found that children of mothers of lower education levels were nearly 3 times more likely to be stunted compared with those of secondary and above levels. It was relatively consistent with the findings from Adama Town, central Ethiopia (10), Wolayta Zone, southern Ethiopia (18), and Uganda (20). The possible explanation for this may be that as women’s level of education increases their awareness of child nutrition, child care practices, healthcare utilization during illness, and immunization service utilization improves, and that in turn affects children’s nutritional status (10,14,17,19).

In addition, the likelihood of being stunted is lower among children of younger employed mothers [AOR = 0.08, 95% CI: (0.01, 0.64)]. It is consistent with a survey report from Wolayta Sodo southern Ethiopia (18). The possible explanation is at a younger age, the number of family size particularly children is limited which enables the mother to give sufficient time to care for her children compared with late age (14,18). On the contrary, as the age of children of employed mother increase the likelihood of being stunted lowers [AOR = 0.37, 95% CI: (0.11, 0.78)]. This was supported by the study conducted in the Wolayta Zone, where the prevalence of stunting was significantly higher in the age group of 6–23 months compared to the older children (14). The possible justification for this may be the intergenerational effect of stunting affects children to be stunted at an early age without intensive intervention.

This study finding also showed that as family income increases (>2,000 ETB), the likelihood of children of employed and unemployed mothers being stunted is lessened. This is in line with the study finding from the Wolayta Zone, southern Ethiopia (18). This indicates increasing income increases food security at the household level, thus decreasing stunting. Moreover, children from low-income households had low access to adequate dietary intake in kinds and amounts and also decreased purchasing power of the family, and a shortage of other important materials and utilities (30).

Children of an unemployed mother who had no exposure to nutrition education were 2.5 times more likely to be stunted compared with those children of mothers who had been exposed to nutritional education. This finding is in line with the findings of a previous study (18). This implies that nutrition information is the best intervention in bringing behavioral change which gives healthy feeding practice that in turn plays a significant role in better nutritional status of children (30). In addition, girls were two times more likely to be stunted compared to the boys. This was supported by a study conducted in a different part of Ethiopia (18, 30, 31) whereas, it contradicted other study findings, that being girls were less likely to be stunted (6, 19, 32). The reason for this discrepancy may be due to unmeasured factors on care giving behaviors of mothers, because of preference of sex in a country like Ethiopia (30).

## Strength and limitation

This is the first comparative study carried out at community level in Gurage Zone, The limitation of this study comprises since the study used the data since birth like breastfeeding practice, Vit-A supplementation, and others, which may confront memory lapse (recall bias), and the Shortage of similar studies limited us to making further comparative discussion.

## Conclusion and recommendations

In our study prevalence of stunting was 21.2%, 95% CI: (17, 25.5) and 28.8%, 95% CI: (23, 33.4) for children of employed and unemployed, respectively. This finding is lower compared with the regional, national, and WHO cut-off point of 40% set for stunting. In this study, maternal education, maternal age, family income, frequency of breastfeeding, and child age had a statistically significant association with stunting among children of employed mothers, whereas nutritional information, maternal education, family income, and sex of the child had a statistically significant association with stunting among children of unemployed mothers, thus nutrition intervention should focus on encouraging women education as it increases the probability of being employed, improving income of family by using different income-generating strategies, and strengthening the existing essential nutrition counseling strategy. Likewise, further research work on the difference between employed and unemployed mothers on stunting status is also recommended to researchers.

## Data availability statement

The raw data supporting the conclusions of this article will be made available by the authors, without undue reservation.

## Ethics statement

Ethical clearance was obtained from the Institutional Health Research Ethical Review Committee, Wolkite University. An official support letter was submitted to those who are concerned. The study, purpose, procedure and duration, rights of the respondents and possible risks and benefits of the study were clearly explained to each participant using the local language. Finally written informed consent was obtained from participants before they participated in the study. The data confidentiality and all COVID-19 pandemic prevention standard precautions were kept.

## Author contributions

All the authors participated in the design, data analysis, drafting, and revising the manuscript, have agreed on the journal to which the manuscript was submitted, approved the final version to be published, and agreed to be accountable for all aspects of the work.
