# Supplementary material for: The relationship between maternal employment and stunting among 6–59 months old children in Gurage Zone Southern Nation Nationality People’s region, Ethiopia: A comparative cross-sectional study
Source: Front Nutr. 2022 Oct 6;9:964124. doi: 10.3389/fnut.2022.964124 (PMC9582235; doi:10.3389/fnut.2022.964124)
Supplement: Supplementary file 1 [file Data_Sheet_1.docx]

Participant information sheet and informed consent form

Good morning/afternoon dear participant!

My name is _________________.

I am working as a data collector for the study being conducted in this community under the title, the Relationship Between Maternal Employment and Level of Stunting and Associated Factors Among 6-59 Months old Children in Gurage Zone, Southern Ethiopia. By Mekiya Ahmed and her co Authors, I kindly request you to lend me your attention to explain the study and be selected as the study participant.

The purpose of the study is to assess the relationship between maternal employment and stunting and associated factors among children aged 6-59 months old. Findings from the study can be used by the study districts to design and develop locally appropriate plans to tackle problems related to these. This may take 30-40 minutes. All the information that you are going to provide me will remain confidential and you don't need to mention your name. For this reason, I kindly request you to give me your sincere and truthful answer.

All of your participation is completely voluntary bases and you have the right to refuse participation. Participation or non-participation and refusal to answer questions will not affect your life. If you have further questions or would like to know the results of this study, please feel free to contact the principal investigator; at the following address.

Principal investigator: Mekiya Ahmed (BSc)

Mobile phone: +251-947696621

E-mail:mekiyaahmed20009@gmail.com

**Consent Form English Version**

I have read all the process and the objective of the study and I have understood the same as written that includes informed about the purpose, advantage, and disadvantage of this study title, the relationship between maternal employment level of stunting and associated factors among children age 6-59 month old I also understood that the research imposes no risk and no compositions would be provided to me. I have been told that if I fill discomfort responding to any of the questions, I fill free to drop them off any time I wish to do so. I have understood the information given and the participation is completely voluntary based.

I have been told that my answers to the questions will not be given to anyone and not expect to write my name. Now I am giving my consent to participate in the study voluntarily. Signature of participant-------------

Could I have your permission to continue?

1. Yes________________2. No______________, Stop and thank the respondent.

Data collector: Name_________ Signature ________________ Date________________

**A structured questionnaire (English Version)**

Part one socio-demographic and economic characteristics

1. .How old are you , Age in year --------------
2. Number of family lives in the HH in number ------------------
3. What is your religion?

1/Muslim

2/Orthodox

3/Catholic

4/protestant

5/ Others specify

1. Marital status

1/married &live together (union)

2/ married live separately

3/Divorced

4/Widowed

5. Never married

1. . Educational status

1/Cant read and write

2/Can read and write

3/primary school

4//Secondary school

5/Collage and above

1. Occupational status of a mother

1 housewife

2 /Government employed

3/privet employed

4/NGO employed

5/daily laborer

6/ merchant

7/Other specify

1. If you are married what is your husband's level of education

1Cant read and write

2/Can read and write

3/primary school

4//Secondary school

5/Collage and above

1. The monthly average income of the family ---------------------- birr per month
2. Occupational status of the husband

1/Job less

2 /Government employed

3/privet employed

4 /NGO employed

5/daily laborer

6/Merchant

7/other specify

1. How is the decision maker for your income

1/ most of the time mother

2 most of the time /Father

3/ Father only

4/ Both together

5/not known /sure

Part two information on characteristics of children aged 6-59 months

1. How old is your child age in month ----------
2. what is the sex of your child

1/ male -----------------

2 female ----------------

13 How many children do you have currently

In number --------------

14.Place of birth

1/at home

2/at health facility

15.Gestational age at birth

1/ less than nine months

2//at 9month

3/greater than nine month

4/do know /not sure

16.Does the child vaccinated for age?

1/yes

2//no

3//I don't know

17. Does the child take Vit A for his//her age

1/Yes

2/No

3//I don't know

18. When did you start to breastfeed the child after birth?

1/ immediately /within one hour

2--------------hour (if less than 24hour)

3----------------day

4/ I don't know sure

19.Did you ever breastfeed the child

1/Yes

2/ No

If yes skip to number 23

20. If the answer of 21 is what is the reason

1/due to illness to mother (including breast)

2/ illness in an infant

3/no breast milk

4/Other Specify

21.Haw Frequent breastfeeds the child per 24 hours--------per 24 hours

22.How many months did you exclusively breastfeed the child------------- months

23.How long did you breastfeed the child------------- month

24.At what age did you start complementary feeding------------ month

25.How many times do you feed your child

1/ one to two times per day

2//three to four times per day

3/ grater four times per day

26.What do you use to feed the child

1/bottle

2/spoon

3/ cup

4/ other specify

27.Has the child had diarrhea in the last two weeks

1/yes

2No

3/ do not know /not sure , If no Skip to 30

28.If yes for 28 how frequent in a day

1/ once

2/ twice

3/ 3-4 times

4//> 4 times

29.who is usually taking care of baby feeding

1/mother

2/sister

3/grandmother

4/servant

5/other specify

Part three information on maternal characteristics

30.Have you visited a health facility for ANC

1/ Yes

2/ no

If the answer is no skip to 34

31.How many times have you visited the facility for ANC

1/ one

2/two

3/ three

4/four and above

32.Do you get nutrition information or feeding practice (during pregnancy immunization postnatal)

1/yes

2/no

Part four environmental conditions

33.What is your main source of drinking water

1/private tap

2private well

3/ public tap

4/ Other Specify

34.How many liters of water is used for domestic propose per day-------------------litter

35.Do you have a latrine

1/Yes/

2/no

36.Type of latrine you use

1/wooden slab /private /

2/concrete slab /private 3//wooden slab /shared /

4/concrete slab /shared

5/shared VIP shared

6//other specify

37. How do you dispose of garbage

1/open field disposal

2/in a pit

3/common pit

4/composing

5/burring

6/other specify

38.Do you have a separate room which is used as a kitchen

1/yes

2/no

Part five information about mothers

39.Employment status

1/ yes (for employed

2/no(for unemployed

If no skip to question number 43

40.If the answer to question number 41 is yes, how many days do you work per week

-------------------day per week

41.How many hours do you work per day

----------- hours per week

42.How much did you earn for this work in birr per month?

-----------------------birr

43.If "no " for question number---------how do you get earning

1/from husband

2/from relatives

3/from help

4/others------------

Anthropometrical measurement of 6 – 59 months old children

Measurement one Measurement two average

Child height in centimeters ------------------------ ------------------------ ------------

Date of birth ------------------------ age ---------------------------------------------
